# Feasibility of introducing compulsory community health fund in low resource countries: views from the communities in Liwale district of Tanzania

**DOI:** 10.1186/1472-6963-13-298

**Published:** 2013-08-08

**Authors:** Boniphace Marwa, Bernard Njau, Joachim Kessy, Declare Mushi

**Affiliations:** 1 Department of Community Health, Tumaini University-Kilimanjaro Christian Medical, College, P.O. Box, 2240, Moshi, Kilimanjaro, Tanzania

**Keywords:** Compulsory community health funds, Community’s views, Feasibility, Acceptability

## Abstract

**Background:**

In 1995, Tanzania introduced the voluntary Community Health Fund (CHF) with the aim of ensuring universal health coverage by increasing financial investment in the health sector. The uptake of the CHF is low, with an enrolment of only 6% compared to the national target of 75%. Mandatory models of community health financing have been suggested to increase enrolment and financial capacity. This study explores communities’ views on the introduction of a mandatory model, the Compulsory Community Health Fund (CCHF) in the Liwale district of Tanzania.

**Methods:**

A cross-sectional study which involved 387 participants in a structured face to face survey and 33 in qualitative interviews (26 in focus group discussions (FGD) and 7 in in-depth interviews (IDI). Structured survey data were analyzed using SPSS version 16 to produce descriptive statistics. Qualitative data were analyzed using content analysis.

**Results:**

387 people completed a survey (58% males), mean age 38 years. Most participants (347, 89.7%) were poor subsistence farmers and 229 (59.2%) had never subscribed to any form of health insurance scheme. The idea of a CCHF was accepted by 221 (57%) survey participants. Reasons for accepting the CCHF included: reduced out of pocket expenditure, improved quality of health care and the removal of stigma for those who receive waivers at health care delivery points. The major reason for not accepting the CCHF was the poor quality of health care services currently offered. Participants suggested that enrolment to the CCHF be done after harvesting when the population were more likely to have disposable income, and that the quality care of care and benefits package be improved.

**Conclusions:**

The CHF is acceptable to the most of study participants and feasible in rural Tanzania as an alternative mechanism to finance health care for the rural poor. Community members are willing to join the scheme provided they are well informed, involved in the design and implementation, and assured quality health care. Strong political will and a supportive environment are key ingredients for the success of the CCHF.

## Background

Finding a way to finance and provide health care for the rural poor in Sub-Saharan African countries is one of the greatest challenges facing governments and other development partners. In Tanzania, health care is primarily financed through government taxation and donor support. Other financing mechanisms include the National Health Insurance Fund (NHIF), social health insurance benefits from the National Social Security Fund (NSSF), private health insurance, user fees (out of payment expenditures) and the Community Health Fund (CHF) [1,2]. The Tanzanian health financing system and its mechanisms are addressed in detail in various government documents, such as the CHF Act 2001, the Health Sector Strategic Plan (HSSP) III 2009 – 2015 and the National Health Policy of 2007 [1,3,4]. The CHF Act of 2001 allows for community contributions in the health sector in order to achieve universal health coverage and address issues of equity in health care services by removing payment hardships [5].

All districts are legally allowed to set by-laws and modes of operationalization, depending on their context. The main sources of health care financing at district level in Tanzania are government block grants, donor funds (health sector basket funds), reimbursements from NHIF and user fees/out of pocket payments [6].

In 1995, Tanzania introduced the voluntary Community Health Fund (CHF) with the aim of ensuring universal health coverage by increasing financial investment in the health sector. The CHF is a pre-payment scheme where members make small payments at regular intervals to decrease the risk of large payments in healthcare fees if a household member becomes ill. The CHF Act of 2001 stipulates that each district sets premiums depending on the local economy and benefits are limited to dispensaries, which are at the lowest level of care [1]. Membership is voluntary and lasts for only one year, but can be renewed. The scheme is limited to 6 core family members. Since 2001 Local government is required by National Health Policy to take care of the vulnerable such as those over the age of 60, the very poor, the disabled, the chronically ill and orphans [4,5].

The HSSP-III (2009– 2015) clearly states several health targets, including the Millennium Development Goals (MDGs) of improving health care delivery by raising the CHF enrolment to 75% coverage and improving health infrastructures [3].

Available evidence shows that voluntary Community Health Fund has had mixed outcome. Positive ones include access to care for members of the scheme, mobilization of funds at district level, raising awareness about pre-payment schemes and its benefits. However, due to poor enrolment voluntary CHF could not create financial protection for the majority of poor and reliable sources of health care financing to reduce government and donor dependency as planned [7,8]. In 2010, 15 years after implementation in various districts in the country, the average enrolment to voluntary CHF stands at six per cent, far below the national target of 75%. However, during the study we found that enrolment to the voluntary CHF which started in 2006 in the Liwale district 17% of the district population slight higher (17% Vs 6%) than the national average. Poor enrolment levels have been associated with the voluntary nature of the scheme, the limited benefit packages such as number of beneficiaries, service restricted to local health facilities and coverage of health conditions. Others include lack of political commitment, weak scheme management and the poor quality of available care, due in part to health staff shortages [2].

Based on the experiences of other nations’ mandatory schemes, such as the Community Based Health Insurance (CBHI) schemes in Rwanda and Ghana, researchers have proposed a compulsory scheme as an alternative in Tanzania to address the weakness of the voluntary CHF. The Compulsory Community Health Fund (CCHF) is a monetary and annual compulsory community prepayment for health care services. If well designed it is believed that the compulsory scheme would be reliable and sustainable [9].

The CCHF would have several advantages: it would pool financial resources from the community, it would make planning and implementation easier, it would increase risk pooling across different socio-economic groups (especially those from informal sectors) and it would eliminate adverse selection. Lastly, it could guarantee access to health care among poorer community members by addressing the existing complex exemption procedures [9,10]. However, it is not yet understood if community members in Tanzania would accept the idea of a compulsory model or what should be considered in the design and implementation of the scheme. Thus, the main objective of this study was to assess the feasibility of introducing the Compulsory Community Health Fund in the Liwale district of the Lindi region of Tanzania.

## Methods

### Design and study area

This study uses a cross-sectional design which employed both quantitative and qualitative methods. Data were collected between May and June of 2011 in the Liwale district of the Lindi region of southern Tanzania. Lindi Region is predominantly rural and the majority of the population lives below the poverty line (less than a dollar per day) and depend on subsistence agriculture, growing cashew nuts as a cash crop and maize, rice and beans as main staple foods. The native ethnic groups are Gingo (70%) and Ndonde (30%) and 80% of the population are Muslim. The literacy rate is approximately 60% with 70% having never gone beyond primary school education [11]. The maternal mortality rate in Liwale is 279/100,000, the infant mortality rate is 98/1000 live births, 46% of deliveries are with a skilled attendant and life expectancy at birth is 58 years. The Liwale district is comprised of 3 divisions, 20 wards and 76 villages. The district population is estimated to be 88,894 [12].

Health facilities at Liwale include the Liwale District hospital, one health centre and 23 dispensaries. As in other district in southern Tanzania, Liwale faces a critical shortage of health workers. According to the 2010 Liwale District Health Reports, the top five health problems in the area are malaria, HIV/AIDS, diarrhea, pneumonia and fevers of known origin. Out of 21,615 households only 3,733 (17%) are enrolled in the existing voluntary Community Health Fund [12]. List of the villages was obtained from the district office. A multistage random sampling using a lottery method was performed in selected study areas. The study sites included the three villages of Kibutuka, Mbaya and Likongowele. The households and individual participants who took part in the quantitative survey were selected using this simple random sampling method.

The minimum sample size (n) estimation for the survey was 400 individuals based on Cochran’s theorem [13]. Survey participants were male and female heads of household both members and non members of the CHF. Those who did not consent and non permanent residents were excluded. The study tools were piloted at Nachingwea District which has a similar socioeconomic profile to Liwale district. Interviews were conducted by the principle investigator and two trained research assistants.

Quantitative data were collected using structured questionnaires administered face-face with closed and open ended questions. The data were cleaned daily, coded and entered into software (SPSS version 16) for analysis. The outcome variables were socio-economic characteristics, knowledge of the existing CHF, status of membership and preferences regarding the CCHF.

Purposive sampling was used to select 33 participants for qualitative interviews who deemed knowledgeable on CHF. Seven participants (key informants) were recruited from the district health office (1), Council Health Service Board Members (1) and four experienced community leaders to obtain their views on the subject due to their involvement in the implementation of CHF. Twenty six participants were involved in focus group discussions. A total of 4 focus group discussions (2 for CHF members, 2 for non-members), comprising of 6 to 7 participants were conducted. The principle investigator moderated the discussions aided by a note taker. The in-depth interviews and focus group discussions were conducted in Swahili which is widely spoken national language. Each session lasted from approximately 45 to 60 minutes. Interviews were digitally audio recorded. The topic guide covered, awareness, knowledge and, experience with the CHF perceptions of the CCHF benefit packages, views on exemption and waivers, premium rates, referral services, feasibility and acceptability of the CCHF, including how best the CCHF could be designed and implemented. Qualitative data, were transcribed verbatim, translated into English, transcribed, coded and analyzed using content analysis.

Ethical approval was granted by Kilimanjaro Christian Medical College Ethical Review Committee and permission was sought from District authorities. Written and verbal consent was obtained from the study participants prior to participation.

## Results

Three hundred and eighty seven people study participants (58% males, 42% females) participated in the quantitative survey; the response rate was 97%. The mean age was 38, with a range of 18 to 90 years. Most of survey participants were subsistence farmers (89.7%) and had received at least some primary education (64.3%). The mean annual income of participants was 200 USD/year (interquartile range of 100 – 400). There was an average of five family members per household (SD, ±3), (range of 1 – 23 per households). The majority of participants had heard about the CHF (89.4%) and had an adequate level of knowledge about the CHF (54.9%). Out of 387, 120 (31%) were active members of the CHF during this study, 228 (58.9%) were neither CHF members nor insured in other health schemes and approximately 39 (10%) had dropped out of the CHF for various reasons, such as poor quality of health care services, unsustainable income or lack of knowledge on the benefits of the scheme. Table 1 presents detailed data on social demographic characteristics. In total 90% of respondents were not aware of the benefit of matching funds from the government.

**Table 1:** Quantitative survey: socio-demographic and economic characteristics of participants

**Variables (n = 387)**	**Number (%)**
***Sex:***	
Male	224 (57.9)
Female	163 (42.1)
***Age (years):***	
20 or less	11 (2.8)
21 – 40	208 (53.8)
41 – 60	136 (35.1)
60 +	32 (8.3)
* Median (IQR), years*	*38 (30-50)*
* Number of household members****(mean ± SD)***	5 ± 2.6
***Education level:***	
No formal education	104 (26.9)
Primary education	249 (64.3)
Secondary education	34 (8.8)
***Occupation:***	
Farmers (Subsitance)	347 (89.7)
Employed	2 (0.5)
Self employed	10 (2.6)
Peasant & employed	28 (7.2)
***CHF membership state:***	
Active member	104 (26.9)
Never been a member	249 (64.3)
Dropped from membership	34 (8.8)
***Acceptability of CCHF***	
In favour of CCHF	216 (55.8)
Did not favour CCHF	171 (44.2)

Qualitative interviews were conducted with 33 participants: (26 FGD) and 7 in-depth interviews with key informant interviews). As shown in Table 2, their ages ranged from 23 to 74 (19 Men; 14 Women). Three key informants had college education and while other 4 participants involved in the (in-depth) interviews had education level ranged from primary to secondary school.

**Table 2:** Qualitative interviews: socio-demographic & economic characteristics of participants

**Variables**	**Total (n =33)**	**Proportions (%)**
***Sex:***		
Male	19	57.6
Female	14	42.4
***Age (years):***		
21 – 40	20	60.6
41 – 60	10	30.3
60 +	3	9
***Education level:***		
No formal education	2	6
Primary education	24	73
Secondary education	4	12
College education	3	(9)
* Number of family members in the household****(mean ± SD)***	5 ± 2.6	
***Occupation:***		
Farmers (Subsistance)	28	84.8
Employed	3	9
Self employed	2	6.2
***CHF membership state:***		
Active member	12	36.4
Dropped from membership	12	36.4
Never been a member	4	12
Others (members of NHIF)	5	15.2

### Acceptability and perceived benefits of the CCHF

In the quantitative survey, of the 387, 221 (57%) accepted the idea of a CCHF while (57%) and did 169 (43.7%) not accept the idea. More than half (56%) of those who accepted (221) said that the CCHF would improve health services because of the increased risk pooling and 68% said that the CCHF would reduce of out of pocket expenditures. Sixty five percent believed that the CCHF would improve the quality of health care services, 94% said it would improve community participation and ownership over the health care and 59% believed that the CCHF would make health care workers more responsible to their clients.

In qualitative interviews, all 14 of the Community Health Fund members and 10 out of 12 of the non members who participated in the focus group discussion, along with the 7 informants were in favour of the compulsory Community Health Fund. The general belief was that the CCHF would enhance universal coverage of Community Health Insurance and would reduce out of pocket expenditure. One informant said *“Community members are used to compulsory contributions for their own development projects such as schools and water projects, why not health insurance, which is so important to all people”.*

Participants had different views on how best the CCHF could be implemented. Out of 387 respondents, 354 (91%) said that community members should be adequately informed and involved in the implementation and 278 (72%) suggested that premiums should be collected at a specific time of year, preferably soon after harvesting. During one qualitative interview, a 38 year old female said “*The payment modalities should be changed and done only once or twice within a year period soon after harvesting when most of the people are likely to have money”.* The proposed payment months were July to August and November to December. The same views were shared by the majority of participants during FGD and interviews with informants.

The FGD members and all informants suggested that primary co-operative societies in the respective wards could be used to collect the premiums from the members with their (members) consent or agreement in their annual or semiannual general meeting. An experienced CHF member commented that “*for those who are not members of cooperatives, local by-laws may be set to enforce the community participation in the scheme as it is done in other programs*”.

### Feasibility of the CCHF

During structured and in-depth interview, several factors were explored regarding the feasibility of the compulsory Community Health Fund. These factors were: premium rates, willingness to pay, payment modalities, scheme packages, implementation strategies and the waiver system. Both qualitative and quantitative findings show that the current premium rates Tanzanian shillings (Tsh 5,000/USD 3.2 per family) for the CHF are not a barrier to enrolment. For example, 232 (60%) of participants said the current premium is affordable while 70 (18%) said the rate is high and might be a barriers. Over 60% of respondents who supported the idea of CCHF proposed that premiums be raised to Tsh. 15,000-20,000 per year per family for comprehensive and referral packages at the district level.

During FGD, in-depth interviews and key informant interviews, respondents expressed willingness to pay higher premium rates if the CCHF would provide comprehensive benefit packages including referral services within and outside the district. Most said the existing rate, which was set in 1995 when the CHF started in Tanzania, did not reflect the current costs of health care services.

Out of 169 (43.7%) who did not agree with the creation of a CCHF, 30 (18%) said the CCHF cannot work in poor settings, 17 (10%) said they did not want to be forced to pay for the current poor health services and 134 (79.3%) rejected because they were not aware of existing community health insurance schemes. One participant in the in-depth interviews who supported the CCHF said, “*Unsatisfactory health care services might be a deterrent to acceptance and implementation of CCHF*”. Similar views were apparent from qualitative interviews which focused reinforced concerns about service quality, specifically inadequate manpower, medical supplies and equipment.

#### Criteria for exemption and waivers

Study participants were asked to give their views on the existing Community Health Fund waiving system and how they would like it to be under the compulsory Community Health Fund. Out of 387 respondents, 124 (32%) favoured exemption/free treatment for the poor and in the open ended question they commented that it is the role of central government to support vulnerable people. One hundred and thirty-one respondents (33.919%) said that poor people should be supported by the local government authority and village council, but only 15 (4%) proposed that poor people should be supported by fellow community members. According to participants, people eligible for waivers include those who do not have any support, like elders, the disabled, orphans, and people with chronic diseases or mental illnesses. Participants proposed that formal procedures to identify vulnerable people must be developed and adhered to. As one key informant said “*this should not be left to local government leaders alone because they are biased*”.

## Discussion

The main objective of this study was to explore community views on introducing the Compulsory Community Health Fund (CCHF) in the Liwale District of southern Tanzania. The findings revealed that the CCHF is feasible and is supported by the majority poor subsistence farmers. The majority believed that the CCHF would result in a strong financial base and improved quality of care. Existing premium rates (Tsh 5,000) under the CHF were not found to be an obstacle, but participants suggested that enrolment CCHF should be done at times when people have income. For successful implementation, the CCHF health care services must be improved and community members must be involved in the design and implementation of the scheme, including the identification of people who qualify for fee waivers and the mode of cost compensation.

In general, the CCHF was accepted by the majority of participants with the expectation that it would reduce catastrophic out-of-pocket expenditures, increase enrolment and promote financial mobilization to improve quality of care. Community views are in line with the goals of risk pooling schemes for achieving universal coverage and access to quality health care services [1,14,15].

### Premium rate affordability

Affordability of premiums or contributions to community health insurance is often mentioned as one of the main determinants of membership [16,17]. Although the majority of participants live below the poverty line, the current premium (Tsh 5000) under the CHF was found to be affordable and this finding is contrary to other studies which found that in poor settings even small premiums can be unaffordable to many households and become a major barrier to enrolment [7]. Community members proposed higher premium rates of Tsh 15,000 to 20,000 (10-15 US dollars) per year. The major concern in the study area is not *how much but when to pay the premiums*. Timing of premium payments is an important determinant to enrolment. Subsistence farmers have seasonal income depending on the harvest and selling of crops. It is during this period when the majority of people are likely to have money available to pay the premium. Similar results were also found in Armenia [18] and in other African settings [19]. The 2002 WHO report on health policy recommended that, depending on the context, there should be flexibility in the payment of premiums [15].

In addition, discussions with key informants revealed that the existing primary co-operatives and other community based organizations can be used as an entry point for the implementation of the CCHF. Therefore, depending on the specific context, premiums rates need to be studied carefully before implementation of the CCHF. This calls for studies to assess communities’ income level, the actual costs of services as well as subsidies from central government and development partners.

### Benefits of package and quality of care

Studies have shown that quality of care can determine its acceptability. One of the major concerns in the study area was poor quality of care. Quality of care in the Lindi Region is characterized by inadequate human resource and inadequate supplies or equipment, including diagnostic facilities. This study demonstrates that the majority of community members are ready to join the CCHF, so long as the quality of care is assured by the government. The general feeling was that there is no need for community to contribute health insurance if the quality of care does not satisfy members’ expectations. A similar observation was reported in study conducted in Tanzania in 2007 [7] and in Guinea-Conakry [20] where lack of quality of care was cited as the most important cause of non-enrolment.

Good quality care requires monetary investment and proper institutionalization and management of health financing system [5]. However, in Uganda there was no evidence that poor quality health care had contributed to low levels of enrolment [21].

Regarding benefits of the package under the Compulsory Community Health Fund, community members proposed that the scheme should be comprehensive and should cover referrals beyond district facilities. This study proposes the CCHF with the removal of co-payments because evidence shows that most community members are unlikely to pay due to the seasonal nature of income. Introducing co- payments to the CHI could be a deterrent to enrollment and could reduce health care utilization due to the lack of this additional fund, which is usually set for very important health care services (for example surgeries, imaging studies, admission fees etc.) than the basic ones [9,22].

Low membership enrolment to the scheme is also associated with inadequate knowledge of the existing scheme among the community members possibly due to poor sensitization programmes at district level. The schemes philosophy was poorly understood by the community and this was also an impingement to scheme expansion. Similar findings were reported in Uganda [21]. For example, ninety percent of respondents did not know about matching funds/grants (a subsidy to the members of the scheme provided by the government for the full amount of the premium contributions). The goal of matching grant is to subsidize the premiums and to sustain the scheme. This implies that, before implementing the CCHF, there must be effective sensitization programs in order to ensure its acceptability and sustainability [14,23].

### Waiver and exception

Inclusion of the poor and vulnerable groups to the Community Health Fund is a major challenge and one of the barriers for enrolment into the scheme. The existing system for implementing the exemption policy under the CHF is weak. The CHF Act of 2001 highlighted that; the power to issue exemptions is vested on the ward health committee after receiving recommendations from the village council. The council has to authorize a CHF card to the exempted and the exempting authorities must seek alternative ways to compensate the fund. The process is complicated and has never been operationalised. People who qualify for waivers are directly referred either to the local facilities or district hospitals and these facilities are not compensated. In Ghana, where the compulsory mode of premium prepayment has been adopted; the health facilities are compensated by alternative mechanism of financing and the enrolment to the insurance scheme has increased by 65%. Studies have suggested that alternatives funds must be sought to compensate waivers before implementing the CCHF scheme [7,19].

The Tanzania National Health Policy (2007) recognizes the waivers and those deserving exemption, such as elders above the age of 60 who cannot afford the charges, vulnerable children, children below the age of 5, pregnant women or those with chronic diseases (like cancer, AIDS, Diabetes, heart diseases, TB etc). Contrary to our expectation that people in rural areas would cherish mutual support, only 4% of the study participants were ready to support their fellow community members who cannot pay for the premium. This paradox can be explained by the fact that, the countries’ policy for the vulnerable is known among the population, and people believe that it is the responsibility of the government to take care of those who qualify to be waived.

Experience from Rwanda where enrollment under compulsory scheme has reached 90% [24] point to the fact that the CCHF is likely to attain universal health insurance coverage within the district, bring more funds to the health sector than user fees, improved quality and availability of health care services. However, it must be noted that the post war recovery interventions might have made it possible for Rwanda to implement the national wide compulsory health insurance schemes [25]. Other benefits include increased social responsibility for health among the community members and increased sense of ownership of health care services, increased equity, reducing inequalities and reducing external dependency for health care financing.

There have now been two decades of the voluntary CHF and other community financing mechanisms like user fees; however their contributions towards improving health care services in the country and attaining some national and global commitments like Vision 2025 and the Millennium Development Goals remain uncertain. These funding sources are difficult to project and collect, and thus unreliable and unpredictable to use in planning, especially in primary health care systems. The CHF funds are sometimes not even mentioned in the local council’s planning budgets. It is not possible to meet current and future health demands with the existing voluntary mechanisms of community financing. We believe that our data support a compulsory scheme [16].

## Conclusion

The CHF is acceptable to the most of study participants and feasible in rural Tanzania as an alternative mechanism to finance health care for the rural poor. For the CCHF to be effective, the following must be in place: ensuring the provision of quality health care, appropriate policy and legal framework, community involvement, improved packages, increased awareness about the scheme and its benefits and leadership commitment. More formative research is needed to explore the potential and feasibility of establishing the CCHF, laws, policy and legal framework must be reviewed to provide supportive environment for the CCHF [26]. Specific areas to be addressed are exemption, premium rates, mode of payment and the management of funds.

### Study limitations

The study had two major limitations. First, the sampled respondents may not been representative of the rest of community, thus limit the generalization of the study. Secondly, inadequate literature on compulsory Community Health Funds might have affected the identification of important variables which could be compared. However, the findings from this study might serve as baseline for similar studies in other settings in and outside the country.

## Competing interests

The authors declare that they have no competing interests.

## Authors’ contributions

BM and DM designed the study, data analysis and drafted the manuscript for publication. BM participated in the data collection. BN and JK were involved in the data analysis and preparation of the manuscript. DM offered scientific advice, inputs and critique during collection and analysis and throughout the preparation of the manuscript. All authors read and approved the final manuscript.

## Pre-publication history

The pre-publication history for this paper can be accessed here:

http://www.biomedcentral.com/1472-6963/13/298/prepub
